# Supplementary material for: A Novel Soy Isoflavone Derivative, 3′-Hydroxyglycitin, with Potent Antioxidant and Anti-α-Glucosidase Activity
Source: Plants (Basel). 2022 Aug 25;11(17):2202. doi: 10.3390/plants11172202 (PMC9460358; doi:10.3390/plants11172202)
Supplement: Supplementary file 1 [file plants-11-02202-s001.zip › plants-1873709-supplementary.pdf]

Supplemental Materials:

Contents

Figure S1. The mass-mass analysis of 3'-hydroxylglycitin (**1**) at the positive mode. A significant signal at *m*/*z* 463.3.

Figure S2. 1D NMR spectrum (^{1}H-NMR, 700 MHz, DMSO-d_6) of 3'-hydroxylglycitin (**1**).

Figure S3. 1D NMR spectrum (^{13}C-NMR, 175 MHz, DMSO- d_6) of 3'-hydroxylglycitin (**1**).

Figure S4. 1D NMR spectrum (DEPT-135, 175 MHz, DMSO-d_6) of 3'-hydroxylglycitin (**1**).

Figure S5. 2D NMR spectrum (^{1}H-^{13}C HSQC, 700 MHz, DMSO-d_6) of 3'-hydroxylglycitin (**1**).

Figure S6. 2D NMR spectrum (^{1}H-^{13}C HMBC, 700 MHz, DMSO-d_6) of 3'-hydroxylglycitin (**1**).

Figure S7. 2D NMR spectrum (^{1}H-^{1}H COSY, 700 MHz, DMSO-d_6) of 3'-hydroxylglycitin (**1**).

Figure S8. 2D NMR spectrum (^{1}H-^{1}H NOESY, 700 MHz, DMSO-d_6) of 3'-hydroxylglycitin (**1**).

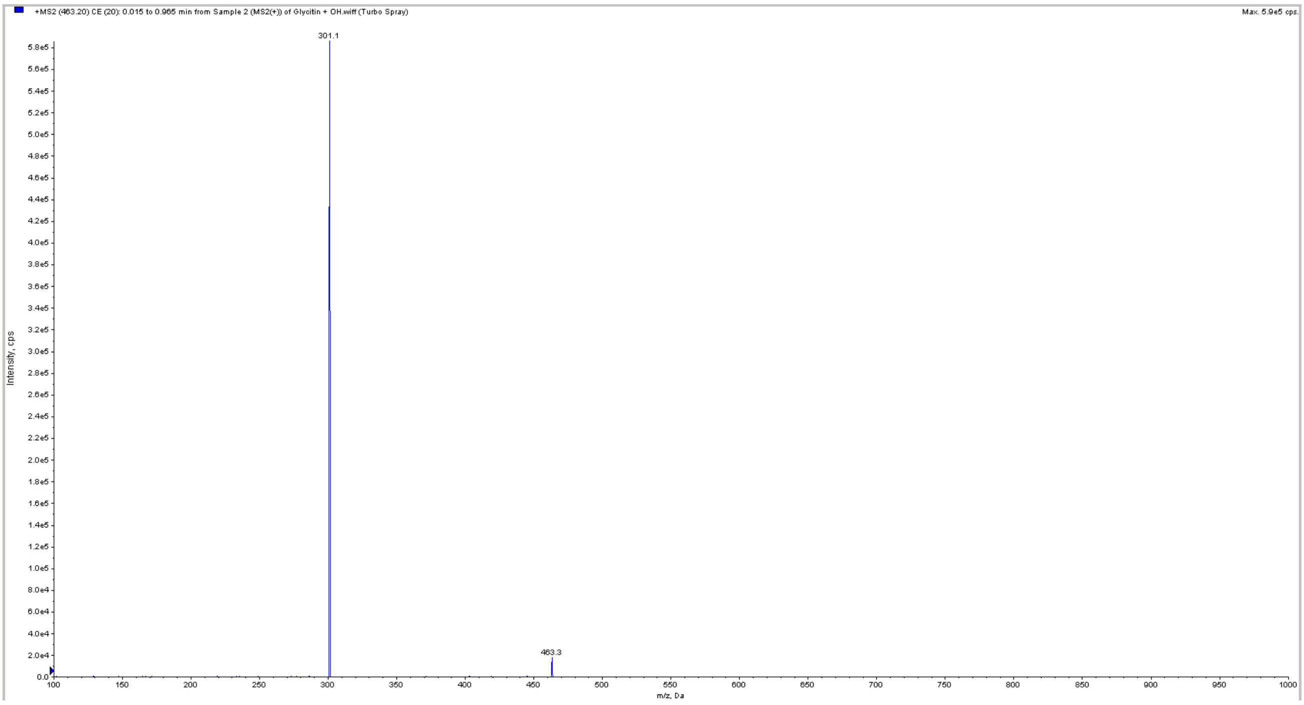

Figure S1. The mass-mass analysis of 3'-hydroxylglycitin (**1**) at the positive mode. A significant signal at *m/z* 463.3.

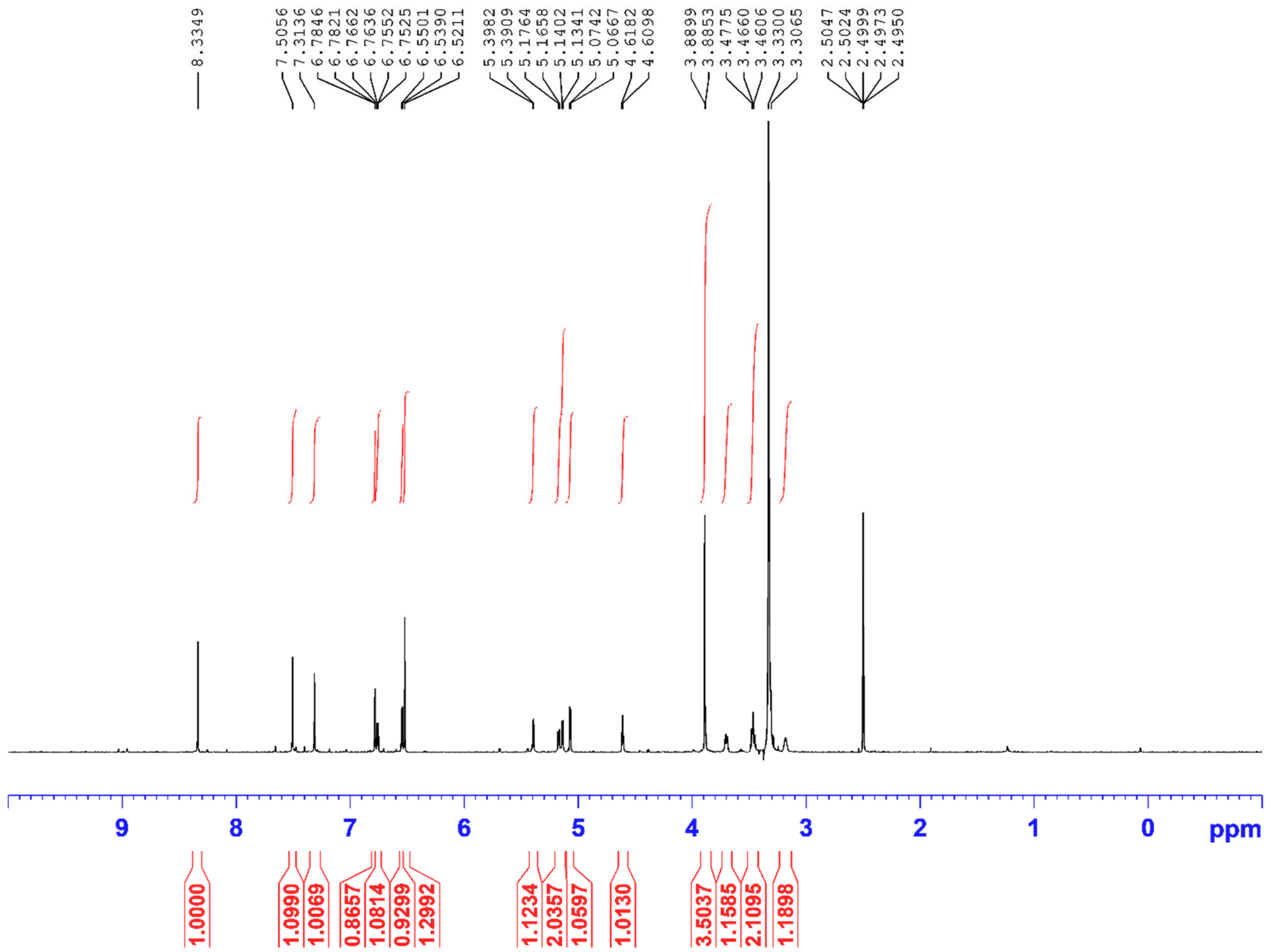

Figure S2. 1D NMR spectrum (^{1}H-NMR, 700 MHz, DMSO-*d*$_6$) of 3'-hydroxylglycitin (**1**).

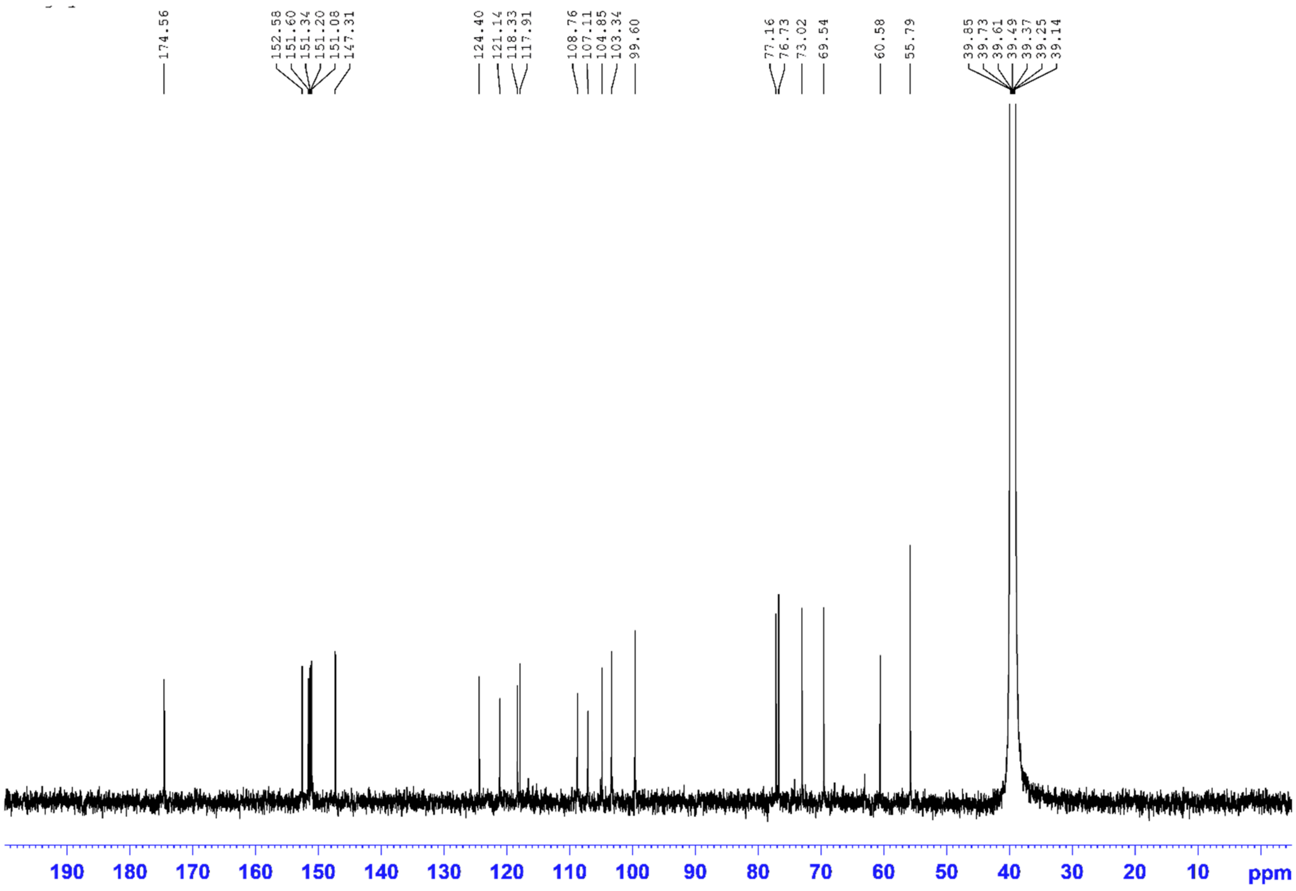

Figure S3. 1D NMR spectrum (^{13}C-NMR, 175 MHz, DMSO- d_6) of 3'-hydroxylglycitin (**1**).

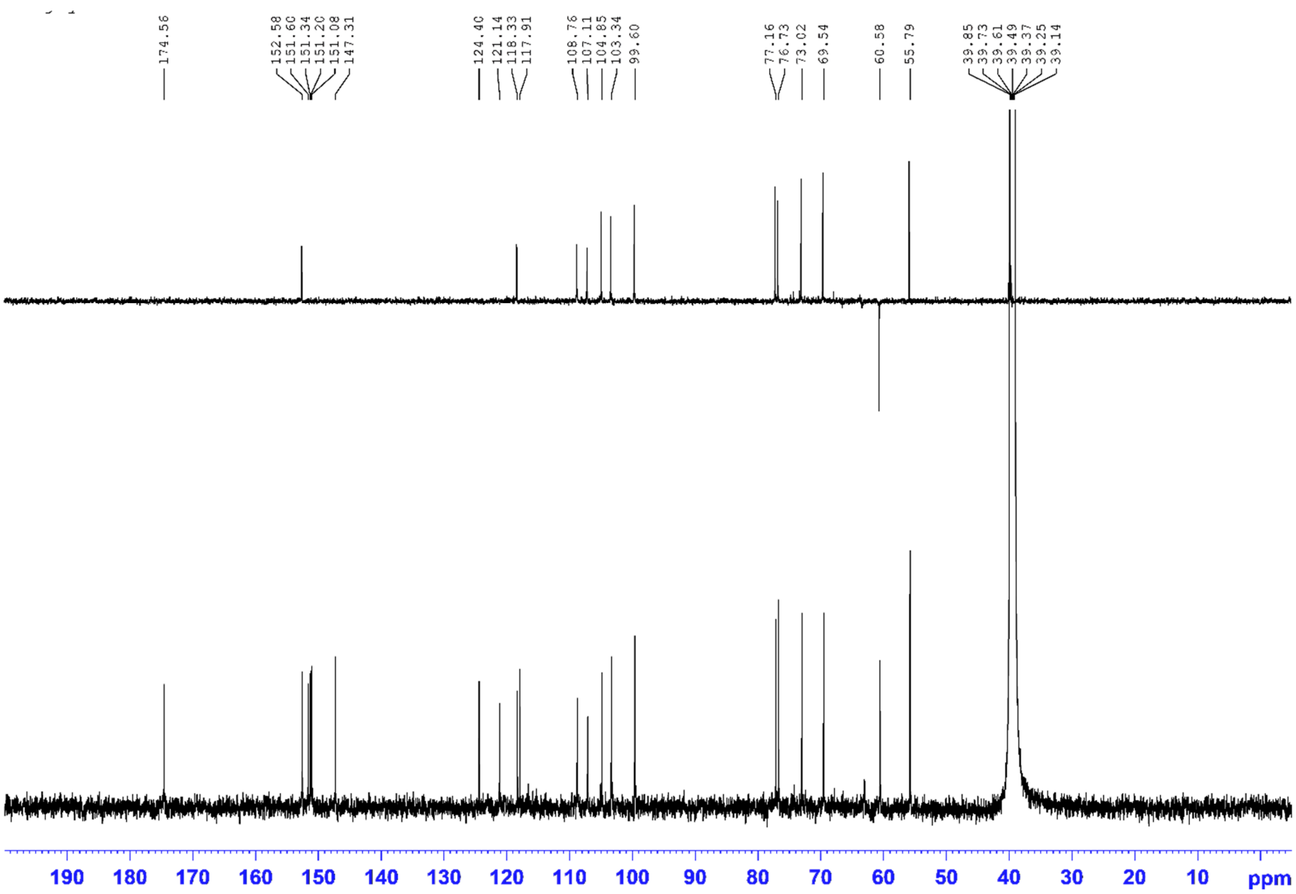

Figure S4. 1D NMR spectrum (DEPT-135, 175 MHz, DMSO-d_6) of 3'-hydroxylglycitin (**1**)

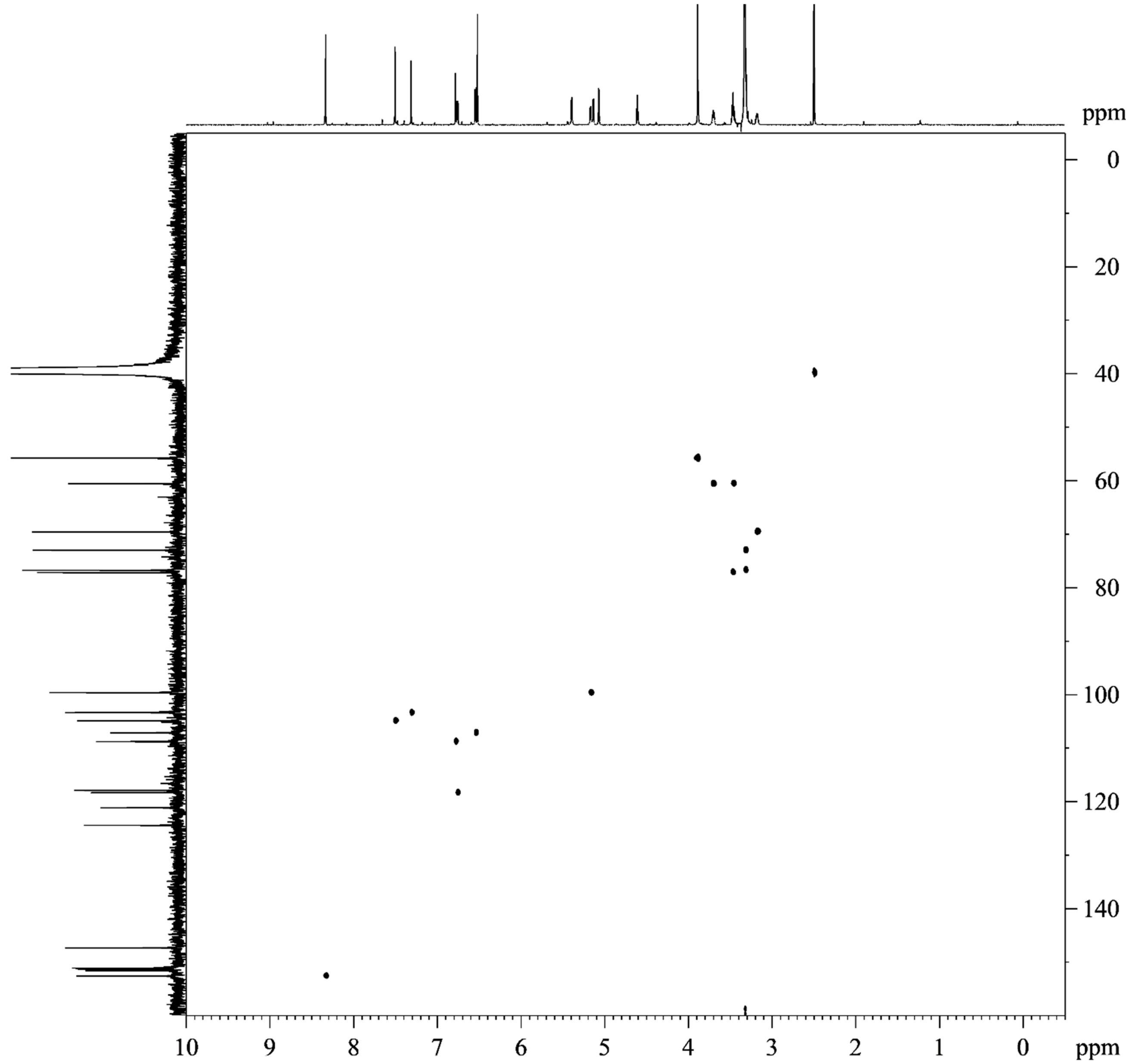

Figure S5. 2D NMR spectrum (^{1}H-^{13}C HSQC, 700 MHz, DMSO-d_6) of 3'-hydroxylglycitin (**1**)

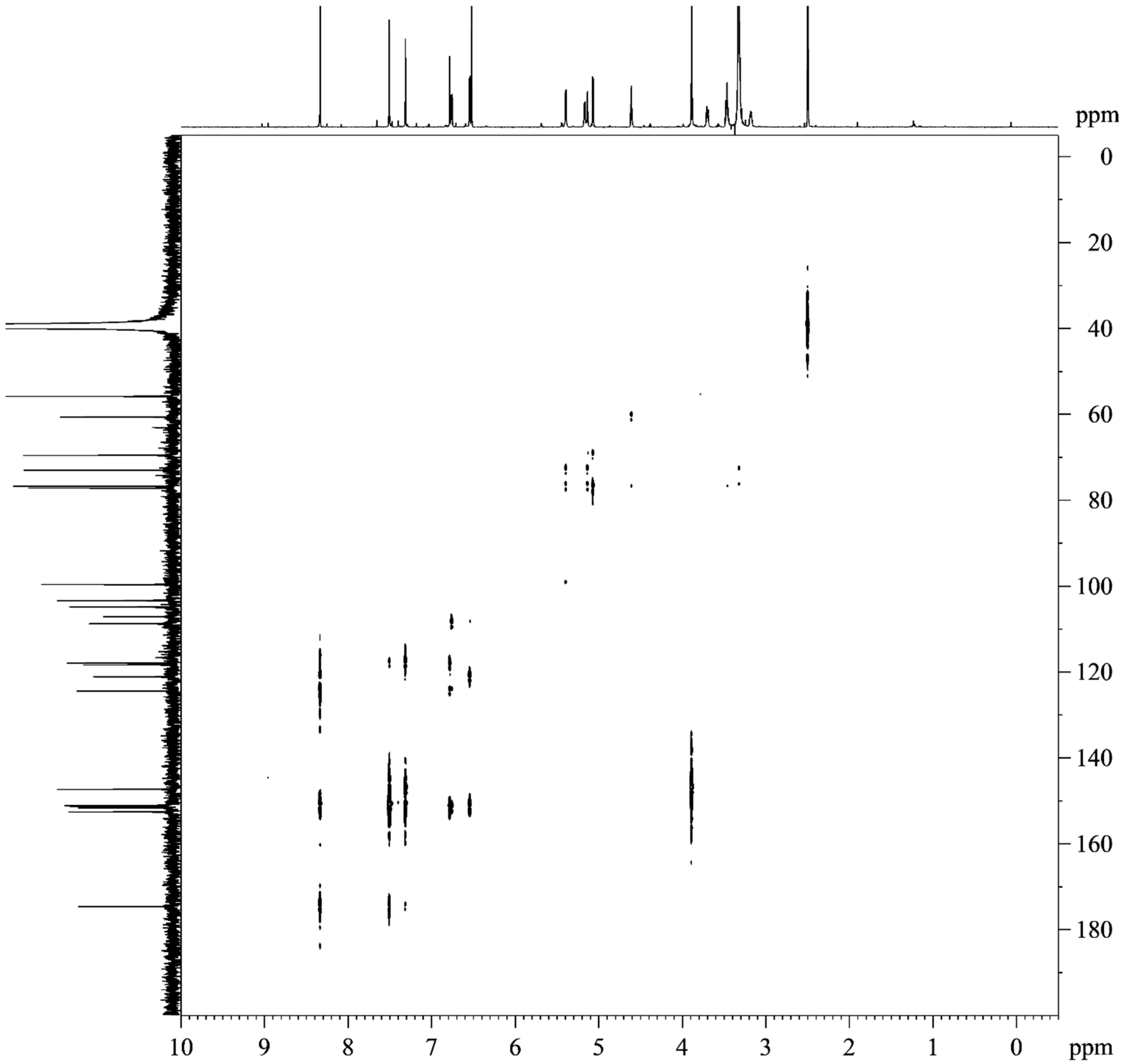

Figure S6. 2D NMR spectrum (^1H-^{13}C HMBC, 700 MHz, DMSO-d_6) of 3'-hydroxylglycitin (**1**)

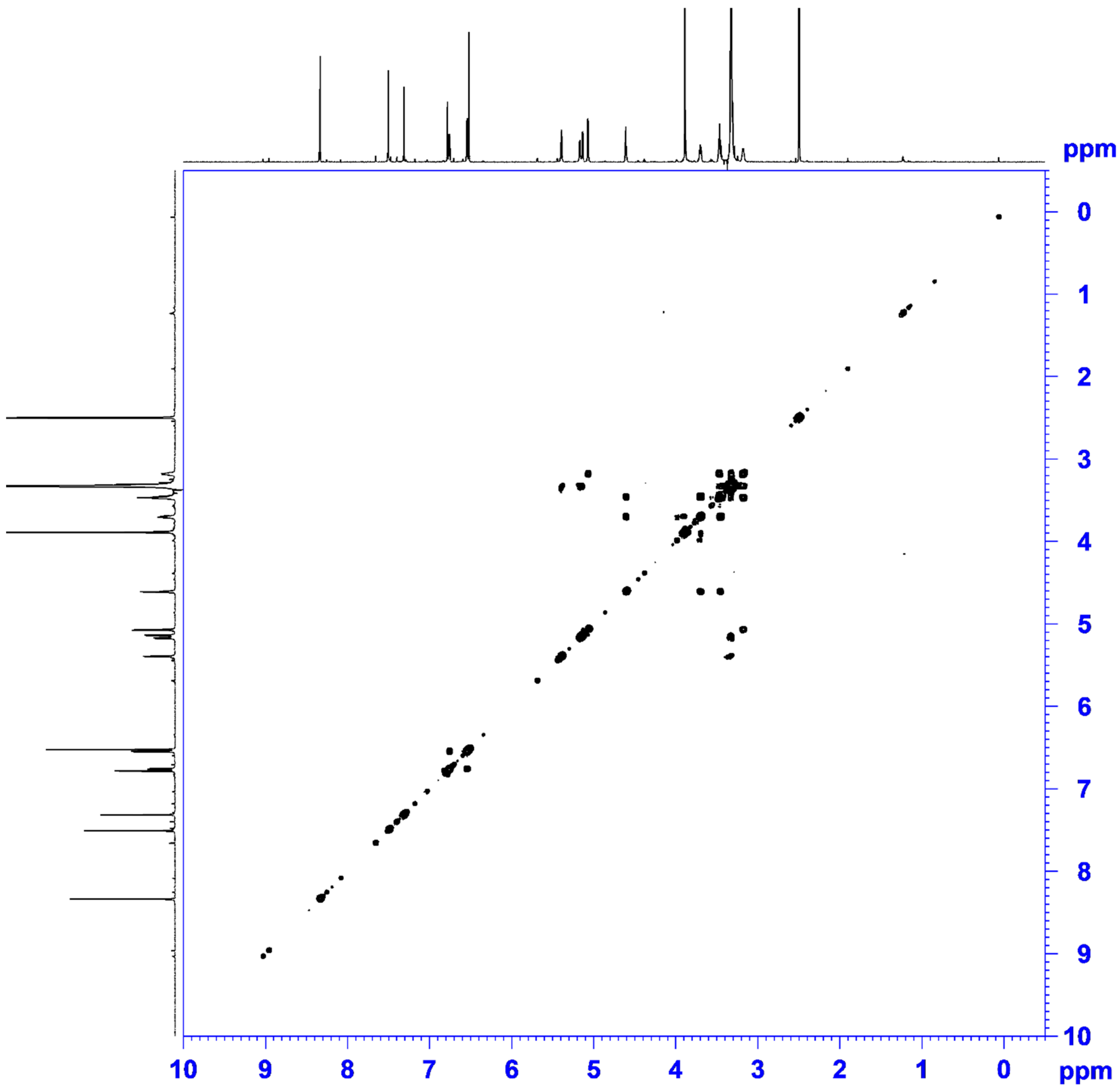

Figure S7. 2D NMR spectrum (^1H-^1H COSY, 700 MHz, DMSO-d_6) of 3'-hydroxylglycitin (**1**)

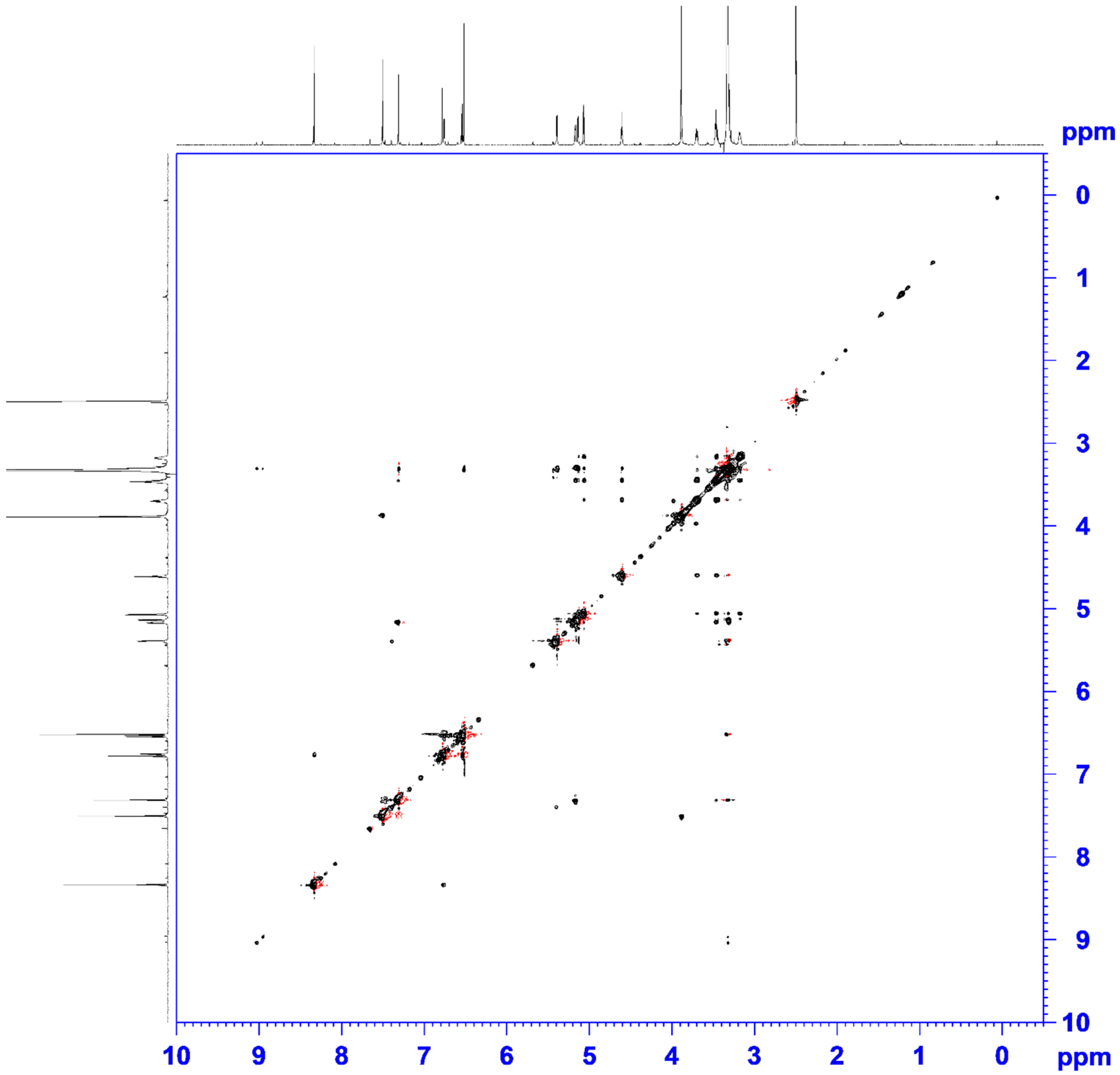

Figure S8. 2D NMR spectrum (^1H-^1H NOESY, 700 MHz, DMSO-d_6) of 3'-hydroxylglycitin (**1**)
